# The Topographical Effect of Optical Zone Diameter in Orthokeratology Contact Lenses in High Myopes

**DOI:** 10.1155/2019/1082472

**Published:** 2019-01-02

**Authors:** G. Carracedo, T. M. Espinosa-Vidal, I. Martínez-Alberquilla, L. Batres

**Affiliations:** Department of Optics II (Optometry and Vision), Faculty of Optics and Optometry, Complutense University of Madrid, Madrid, Spain

## Abstract

**Purpose:**

To evaluate the effect of the optical zone diameter (OZ) in orthokeratology contact lenses regarding the topographical profile in patients with high myopia (−4.00 D to −7.00 D) and to study its effect over the visual quality.

**Materials and Methods:**

Twelve patients (18 eyes) were fitted with overnight orthokeratology (OrthoK) with a randomized 6 mm or 5 mm OZ lens worn for 2 weeks, followed by a 2-week washout period, between both designs. Keratometry (K) readings, optical zone treatment diameter (OZT), peripheral ring width (PRW), higher-order aberrations (HOA), high (HC) and low contrast (LC) visual acuity, and subjective vision and comfort were measured at baseline and after 2 weeks of OrthoK lens wear of each contact lens.

**Results:**

No significant differences were found between any measurements for the same subject at both baselines (*p* value > 0.05). There was no difference between OZ lens designs found in refraction, subjective vision or comfort, and HC and LC visual acuity. Contrast sensitivity was decreased in the 5 mm OZ lens design compared with 6 mm OZ design (*p*-value < 0.05). 5 mm OZ design provoked a greater flattening, more powerful midperipheral ring and 4^th^-order corneal and total spherical aberration than the 6 mm OZ design, being statistically significant after 7 days, for corneal aberration, and 15 days, for corneal and total, of wearing the lens (*p*-value < 0.05). The OZT obtained were 2.8 ± 0.2 mm and 3.1 ± 0.1 mm for 5 mm and 6 mm OZ design, respectively (*p*-value < 0.05). Regarding PRW, the 5 mm OZ design had a wider ring width in both the nasal and temporal zones (*p*-value < 0.05).

**Conclusions:**

A smaller diameter optical zone (5 mm) in orthokeratology lenses produces a smaller treatment area and a larger and more powerful midperipheral ring, increasing the 4^th^-order spherical aberration that affects only the contrast sensitivity but without differences in visual acuity and subjective vision compared with a larger OZ diameter (6 mm).

## 1. Introduction

Currently, orthokeratology (OrthoK) has become a clinically reliable and effective method to correct refractive errors using specialty gas permeable contact lenses [1–3]. OrthoK contact lenses are worn overnight and removed in the morning upon awakening, and they provide great quality of vision [3]. A strong advantage with OrthoK is that it is a reversible procedure, so when the use of the lens ceases completely, the cornea recovers to its initial physiological state [4–6].

The tear film under the OrthoK lens reshapes the cornea in closed eye conditions by applying a positive push pressure over the central cornea and a negative pull pressure in the midperiphery [2]. This produces corneal flattening in the central treatment zone to reduce corneal power for myopia correction and corneal steepening, creating a plus power, in the midperiphery. Previous studies have shown changes in corneal thickness with the epithelium thinning in the central zone and thickening in the midperiphery [5, 7, 8].

Orthokeratology was originally prescribed for adults to correct myopia during the day without glasses or contact lens wear [9]. Currently, the majority of OrthoK lenses are being prescribed for myopia control. Several studies have demonstrated the efficacy of OrthoK in slowing down axial length elongation [10–15]. Together with soft contact lenses and pharmacological treatments such as atropine, OrthoK is considered one of the most effective treatment options for slowing myopia progression [15]. The mechanism of action by which OrthoK lenses slow myopia progression is not completely known. Peripheral defocus, as described in animal models is the most common theory [16–18]. Myopic children are found to have a peripheral hyperopic defocus. OrthoK lenses reshape the midperipheral cornea to steepen, which increases the power, causing a peripheral myopic defocus. This could be a factor in slowing down the axial growth and myopia progression [19–21].

Together with peripheral defocus, higher-order aberrations have been associated with myopia control, mainly 4^th^-order spherical aberration and coma [22, 23]. Faria-Ribeiro et al. described that higher-order aberrations are associated with larger pupil diameters as well as the effect on myopia control, potentially as a result of a larger retinal area exposed to the peripheral myopic defocus [24]. Many studies have shown that higher-order aberrations increase significantly after OrthoK treatment, even in successful fittings [23, 25].

Thus, newer OrthoK lens designs are trying to increase the peripheral myopic defocus and take into account pupil size dependence in higher-order aberrations. These lenses are being developed with a smaller optical zone (OZ) in attempts to achieve a smaller treatment zone and a steeper, more power midperipheral ring closer to the pupil. There are few studies published that have studied the effect of lens design over the cornea [26–28]. The purpose of this study was to evaluate the topographical effect of changing the optical zone (OZ) diameter in OrthoK in patients with high myopia (−4.00 D to −7.00 D) and to study the effect over visual quality. This will enhance our understanding of the effects of OrthoK lens design over a topography profile.

## 2. Materials and Methods

A prospective, longitudinal, and randomized pilot study has been conducted. Twelve healthy subjects (18 eyes, 8 women and 4 men) were recruited in the Faculty of Optics and Optometry (Complutense University of Madrid, Spain). The mean age of patients was 25.01 ± 6.91 years (range 18–27 years old) and mean spherical refractive error −4.72 ± 0.36 diopters (D) (range −4.00 D to −7.00 D). Each subject signed an informed consent after the study protocol, and risk and benefits of the treatment were explained. Participants were free to leave the study at any time without any reason. This study obtained ethical approval from the Ethical Committee of the Complutense University of Madrid and followed the tenets of the Declaration of Helsinki [29].

Inclusion criteria were myopia between −4.00 diopters (D) and −7.00 D and with astigmatism less or equal to −1.50 D. Exclusion criteria were history of ocular disease or systemic disease that could affect visual system or pregnancy. Contact lens wearers were asked to stop wearing their habitual contact lens one week before the examination day. All subjects were fit with Paragon CRT™ contact lenses (Paragon Vision Sciences, Gilbert, AZ) in HDS 100 material (paflufocon D, Dk = 100 barrer) according to manufacturer guidelines.

The study was divided into two phases, in which the patient used two different types of OrthoK lenses: a lens with 6 mm OZ diameter and another lens with 5 mm OZ (Table 1). Patients were randomly chosen to start with one of the lens designs and wear the lens consistently for 15 days and 14 nights [30]. This was then followed by 15 days of washout time without any contact lens wear in an attempt to allow the cornea to return to its physiological baseline [31]. Then the patient would resume contact lens wear for 15 days with the other lens design. Investigators made sure to give proper instruction to the patient of handling and care of lenses, including application and removal of OrthoK and contact lens solution care system.

Refraction without cycloplegia, high (HC) and low (LC) contrast uncorrected visual acuity (UCVA), HC and LC best-corrected visual acuity (BCVA), contrast sensitivity, corneal topography, anterior corneal and total wavefront aberration, and Visual Analogue Scale (VAS) questionnaire were performed. All measurements were performed at baseline for pretreatment records (PRE), at 1 day (after the first night of lens wear), at 7 days, and at 15 days for both lens designs, except for total wavefront aberration, contrast sensitivity, and LC BCVA and UCVA. All measurements were taken early in the morning, so that the patient had slept with the lenses for at least 6 hours. A slit lamp examination was performed in all visits to verify the ocular surface integrity.

**Figure 1:**
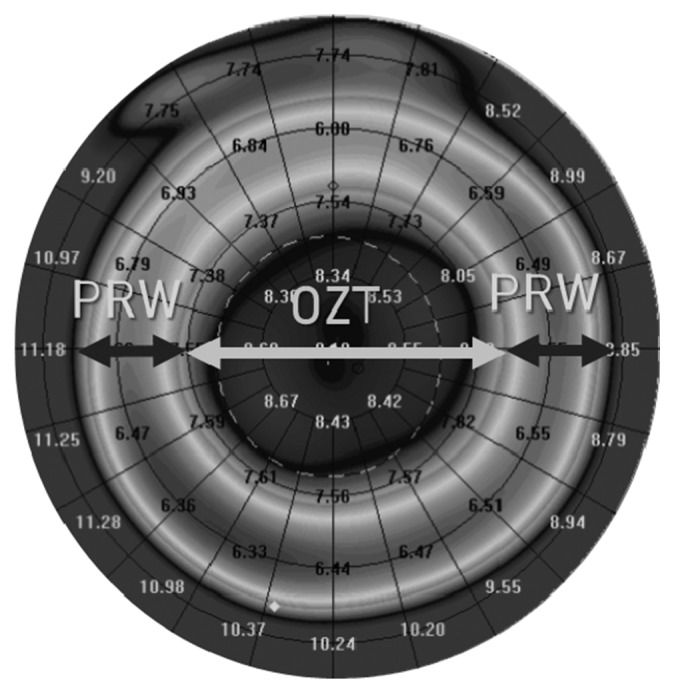
Tangential topography map to show the optical zone treatment (OZT) and peripheral ring width (PRW), both parameters analyzed in this study.

Corneal topography was taken with the Scheimpflug camera system, Oculus Pentacam (Oculus, Wetzlar, Germany). Parameters obtained with Pentacam included flat keratometry (flat k), steep keratometry (steep k), and corneal radii in *X*-axis from 4 mm of distance to the apex in both nasal and temporal directions. In addition, optical zone treatment diameter (OZT) and peripheral ring width (PRW) were defined. The OZT was defined as the central zone of corneal flattening from baseline after OrthoK wear. Margins of this zone were marked from comparison map topography data, considering the OZT diameter where the difference between before and after orthokeratology wear was zero. The corneal zone where the corneal radius was steepened during OrthoK lens wear from baseline reading was defined as PRW. PRW margins were considered as the width between OZT margins and where the corneal radius returns (from steepening) the same before and after OrthoK lens wear. PRW was measured in the nasal and temporal zones of *X*-axis (Figure 1).

Subjective refraction was performed based on the patients' current spectacle prescription, and a fogging method was created for obtaining the final subjective refraction. The astigmatism was adjusted by crossed cylinder technique. The main objective was to find the BCVA with the maximum positive sphere. UCVA and BCVA were measured monocularly in photopic luminance conditions (85 cd/m^2^) using the ETDRS test form Chart Display VX24 (Visionix Ltd., Visionix-Luneau Technologies, Chartres, France) with HC (contrast level 100%) and LC (contrast level 10%) at 4 meters. LC BCVA and UCVA were measured at baseline and 15 days after each lens design; HC BCVA and UCVA were measured at all visits. Contrast sensitivity was measured using the Pelli-Robson test at 1 meter, in which spatial frequency corresponds to 1 cycle per degree. VX110 (Visionix-Luneau, France) was used to determine the changes in 4^th^-order spherical aberration for corneal and total spherical aberrations.

### 2.1. Statistical Analysis

Statistical analysis was performed using the SPSS Statistics 23 software (IBM, Chicago, Illinois, USA). Sample size calculations were performed with statistical software Granmo 6.0 (Institut Municipal d'Investigació Mèdica, Barcelona, Spain). A statistical power of 80% was considered. Considering the horizontal corneal radius as the main variable, with an accepted two-sided statistical significant threshold of 0.05 and a risk of 0.20, for a standard deviation of 0.06 units to the mean and in order to detect a difference of 0.05 units, at least 12 subjects were needed to find statistically significant differences. Normality of samples was analyzed using the Kolgomorov–Smirnov test. To analyze the differences between the baseline and additional visits and also between both lens designs in the same visit, a Student's *t*-test for paired samples has been used. Repeated measures ANOVA test was performed to evaluate the trend of the different parameters tested during the study. Results are shown as mean ± standard deviation, and a statistical significance of 95% was established (*p* < 0.005).

## 3. Results

All patients completed the study without drop outs. No significant differences were found between any baseline measurements for the same subject in the two different lenses (*p* value > 0.05; Student's *t*-test for paired samples). Refraction improved from the first day of OrthoK lens wear (*p* value < 0.05; Student's *t*-test for paired samples), being −2.51 ± 0.35 D and −2.87 ± 0.97 D for 1 day of wear and −0.31 ± 0.80 D and −0.35 ± 0.51 D at 15 days of wearing for 5 mm OZ and 6 mm OZ designs, respectively. No differences in refraction were found between designs (*p* value > 0.05; Student's *t*-test for paired samples).

Table 2 summarizes the mean values and standard deviations of visual acuity and contrast sensitivity obtained during the baseline and follow-up visits. HC UCVA was statistically lower than HC BCVA after one day of orthokeratology lenses for both OZ designs (*p* value < 0.05; Student's *t*-test for paired samples). However, after seven days of wearing, both designs showed HC UCVA improvement, reaching the BCVA at the baseline (*p* value > 0.05; Student's *t*-test for paired samples). In addition, no differences were found between designs for any visit studied (*p* value > 0.05; Student's *t*-test for paired samples). Regarding LC visual acuity, 6 mm OZ lenses showed a decrease in LC UCVA after 15 days of wear compared with LC BCVA at the baseline (*p* value = 0.004; Student's *t*-test for paired samples). Meanwhile, with the 5 mm OZ design, LC UCVA was slightly worse than LC BCVA but not statistically significant (*p* value > 0.05; Student's *t*-test for paired samples). As HC visual acuity, no differences were found for LC visual acuity between designs for any visit studied (*p*-value > 0.05; Student's *t*-test for paired samples).

The contrast sensitivity (CS) was also evaluated. No differences were found between orthokeratology lens designs (*p* value > 0.05; Student's *t*-test for paired samples). However, during 5 mm OZ wearing, CS statistically decreased (*p* value = 0.003; Student's *t*-test for paired samples). While the 6 mm OZ wearing provoked a slight increase in CS, this was not statistically significant (*p* value = 0.195; Student's *t*-test for paired samples).

**Figure 2:**
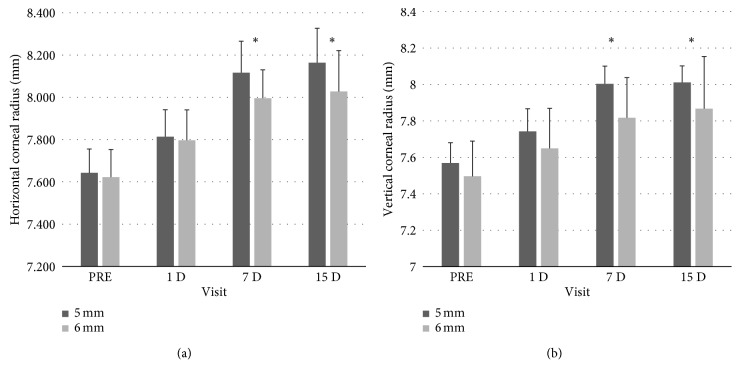
Horizontal and vertical corneal radius in the different visits with 5 mm and 6 mm optic zone lenses ∗ 5 mm OZ vs. 6 mm OZ (*p* value < 0.05; Student's *t*-test for the related samples).

Regarding corneal topography, Figure 2 shows central corneal flattening after orthokeratology lens wear from the first day, being statistically significant for both vertical and horizontal radii in all visit evaluated (*p* value < 0.05; Student's *t*-test for paired samples). The 5 mm OZ design had a statistically significant greater flattening than 6 mm OZ design after 7 and 15 days of wearing the lens (*p* value < 0.05; Student's *t*-test for paired samples). The horizontal and vertical corneal radius flattening differences between lens designs after 15 days of wearing were 0.13 ± 0.02 mm and 0.14 ± 0.06 mm, respectively.

**Figure 3:**
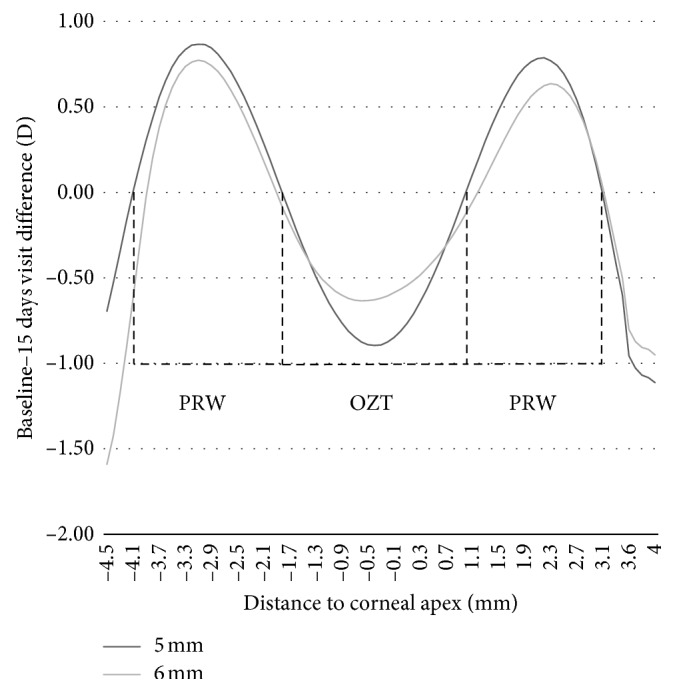
Mean corneal radius differences along the horizontal axis between baseline (before orthokeratology wearing) and day 15 for 5 mm and 6 mm OZ lens designs (BASELINE-15 d). Standard deviation has been removed for a better profile comprehension. Negative values mean flattening and positive values mean steepening. Complete data are show in Table 4. OZT: optical zone treatment; PRW: peripheral ring power.

The keratometric or topography profile of the cornea for each OZ lens design before 15 days of lens wear is displayed in Figure 3. These profiles were created by comparing baseline keratometry and the keratometry measurements after 15 days of wear in OrthoK. The 5 mm OZ design produced greater central flattening and greater midperipheral steepening than 6 mm OZ design for all follow-up visits (*p* value < 0.05; Student's *t*-test for paired samples).  Table 3 shows the changes in corneal radii at different points to the apex in *X*-axis. The treatment size of the 5 mm OZ lens design was 2.8 ± 0.2 mm and 3.1 ± 0.1 mm with 6 mm OZ lens design, with a statistically difference (*p* value = 0.024; Student's *t*-test for paired samples) (Table 4). Regarding PRW, a statistical significance difference was found for the nasal and temporal zone between both lens design, being wider for 5 mm OZ design (*p* value = 0.037 and *p* value = 0.049 for nasal and temporal, respectively; Student's *t*-test for paired samples). These differences provoke a very different keratometric profile in the *X*-axis, with the changes between central cornea and peripheral ring more abrupt. This demonstrates that for 1.00 D of anterior corneal power difference (between the center and the periphery), the 5 mm OZ design measured 1.3 mm to the center. The 6 mm OZ design is a wider measurement of 2.1 mm. Likewise, to reach a power difference of 1.50 D from the center and the periphery, the 5 mm OZ lens design was measured at 2.1 mm and 2.4 mm for the 6 mm OZ designs.

Corneal and total spherical aberration with a 5 mm pupil diameter was also evaluated. Both 4^th^-order corneal and total spherical aberrations (Z12) had a statistical significant difference for all follow-up visits compared with baseline for both OZ lens designs (*p* < 0.05; Student's *t*-test for paired samples), trending towards greater positive corneal spherical aberration. Comparing both designs, no statistical differences were found at baseline and wearing OrthoK after 1 day; however, the 5 mm OZ design showed greater positive spherical aberration than the 6 mm OZ design after 7 and 15 days of wearing the lens, representing a statistical significant difference for both corneal and total spherical aberrations (*p* < 0.05; Student's *t*-test for paired samples). See Table 5.

**Figure 4:**
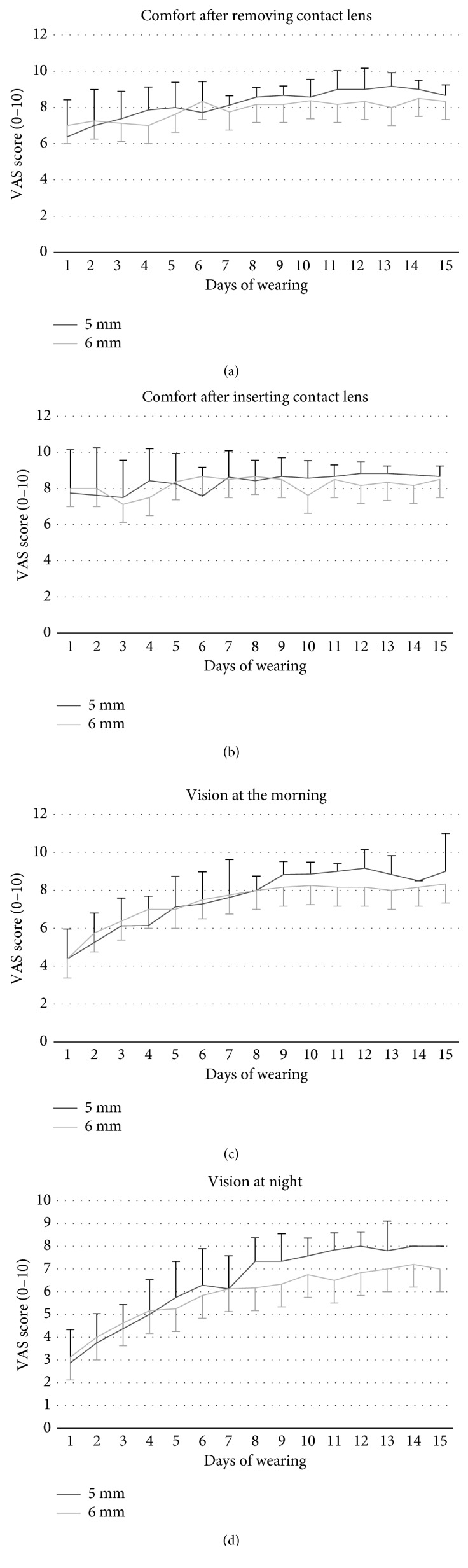
Subjective comfort and visual ratings obtained in the Visual Analogue Scale (VAS) questionnaire with a 5 mm and 6 mm OZ lens design.

Regarding VAs, no significant differences were found between either OrthoK OZ designs for subjective comfort and vision (*p* > 0.05; Student's *t*-test for paired samples). However, less corneal staining was observed in 37.5% of eyes with 6 mm OZ diameter compared to 62.5% with 5 mm OZ diameter (Figure 4).

## 4. Discussion

The current study had the aim to analyze the effect of different OZ diameters of OrthoK lenses over the topography profile in high myopia patients. The 5 mm OZ lens design is able to generate a greater midperipheral curvature and central flattening than 6 mm OZ design. This profile difference means that smaller OZ produces a narrower treatment area and a wider and steeper peripheral ring, closing the steepening ring to the pupil center.

Currently, the scientific literature regarding the effect of lens design, and in particular the optical zone diameter, in OrthoK lenses is very weak. Few studies have been published in this interesting research field [26–28]. Kang et al. described that decreasing the OZ of OrthoK lenses produces minimal effects in corneal molding and in peripheral refraction in low myopia (−1.00 D to −4.00 D) [26], opposite than what was discovered and described in this manuscript. The main difference between these studies could be the initial amount of myopia treated and also the lens design. Kang et al. recruited patients from −1.00 D to −4.00 D, while in the current study, the myopia range was from −4.00 D to −7.00 D.

Changes in the topographic profile, including a wider, steeper midperipheral ring, and closer to the pupil center, has been hypothesized as an important factor in improving the efficacy of myopia control with OrthoK lenses [24, 27, 32]. In a study published in 2016, Kang et al. suggested that inducing greater degrees of myopic defocus on the peripheral retina, more than what is habitually experienced in a typical OrthoK lens, may be required for effective myopia control [33]. On the other hand, another study concluded that different contact lens designs for OrthoK do not provoke significant differences in peripheral refraction [28]. The ring of peripheral curvature closer to the pupil center with 5 mm OZ design and with greater midperipheral corneal power could improve the efficacy in myopia control, although there are no studies that corroborate this hypothesis. A limitation of the present study is that it was not possible to measure peripheral refraction in these patients recruited, and therefore, it is not possible to assert that the topographic profile changes with orthokeratology have impact in myopia progression.

Visual quality is another factor to consider when patients are fit in OrthoK lenses. There are a lot of studies published describing a decrease in visual quality during OrthoK treatment [25, 34–37]. OrthoK changes refraction by flattening the central cornea and subsequently steepening the midperipheral cornea [2]. It would be expected that 5 mm OZ design shows faster refraction correction, but the results of this study do not show differences between both OZ designs. In addition, for HC and LC visual acuity, findings were in the same way, no differences between lens designs. Nevertheless, contrast sensitivity was only decreased with 5 mm OZ orthokeratology design wearing. Hiraoka et al. described a significant decrease of contrast sensitivity after wearing OrthoK lenses, but there is no scientific literature published regarding the effect of OZ diameter over the contrast sensitivity [38, 39]. Liu et al. found that contrast sensitivity decreases after orthokeratology treatment, being alleviated by a larger treatment zone diameter and a smaller lens decentration [34]. In addition, Jung et al. described that tinted contact lenses significantly increased ocular aberrations and decreased contrast sensitivity in function of pigment-free optical zone diameter decreasing [40], taking into account that the differences in 4^th^-order spherical aberration found between both designs studied could be the most probable reason to observe lower contrast sensitivity with smaller OZ diameter in OrthoK.

Given the nature of OrthoK treatment which produces a molding on the corneal surface, it is expected that changes occur with corneal aberrations, and consequently total aberrations [41, 42]. Higher-order aberrations are directly influenced by pupil diameter [24], affecting the mesopic and scotopic visual quality. The results obtained in this study, with both OZ designs, agree with previous studies [24, 25, 37, 43, 44]. As would be expected, the aberrations are greater with the 5 mm OZ design than with the 6 mm OZ design, due to the differences in the topography profile achieved with each lens design. This fact could explain the lower contrast sensitivity with the smaller 5 mm OZ design. However, this loss of visual quality was not extrapolated to change subjective visual satisfaction. Both contact lens designs showed similar scores, even with a slight positive trend in 5 mm OZ design for vision at night compared with the 6 mm OZ lens. This means that an aberrometric alteration occurs with this change in OZ design, even in successful fitting.

In conclusion, a smaller diameter OZ in OrthoK lenses produces a smaller treatment area and a larger and more powerful midperipheral ring, increasing the 4^th^-order spherical aberration that affects only the contrast sensitivity, but without differences in terms of visual acuity and subjective vision, compared to a lens design with a larger OZ diameter. More studies are needed to understand if these outcomes only represent corneal modifications or if a smaller treatment zone and more powerful midperipheral ring, closer to the pupil center, play an important role in increasing the efficacy of myopia control in an OrthoK lens.

## Figures and Tables

**Table 1:** Contact lens parameters used during the study.

Parameter	6 mm OZ lens design	5 mm OZ lens design
Manufacturer	Paragon vision sciences (Gilbert, AZ)	Paragon vision sciences (Gilbert, AZ)
Material (USAN)	Paflufocon D	Paflufocon D
Brand	Paragon CRT	Paragon CRT
Back surface geometry	Sigmoid geometry	Sigmoid geometry
Front surface geometry	Mirrored with anterior surface	Mirrored with anterior surface
Overall diameter	10.50	10.50
Optic zone diameter	6.00	5.00
Reverse curve (RZD) width	1.00	1.00
Landing curve (LZA) width	1.00	1.50
Power fitted (D)	+0.50	+0.50
Back optic zone radius (mm)	7.90 to 8.90	7.90 to 8.90

**Table 2:** High‐ and low‐ contrast visual acuity during orthokeratology wear at different visits.

Parameter (mean ± SD)	HC VA (logMAR)			LC VA (logMAR)			CS (logMAR)		
Optical zone diameter (mm)	5 mm	6 mm	*p* value	5 mm	6 mm	*p* value	5 mm	6 mm	*p* value
PRE (BCVA)	−0.03 ± 0.12	-0.01 ± 0.11	0.822	0.18 ± 0.09	0.17 ± 0.11	0.734	1.84 ± 0.16	1.59 ± 0.66	0.164
1 day (UCVA)	0.52 ± 0.36	0.38 ± 0.30	0.138	—	—		—	—	—
7 days (UCVA)	0.02 ± 0.30	−0.08 ± 0.16	0.176	—	—		—	—	—
15 days (UCVA)	−0.02 ± 0.12	−0.04 ± 0.09	0.730	0.23 ± 0.33	0.38 ± 0.16	0.252	1.47 ± 0.59	1.67 ± 0.05	0.210

D: diopters; mm: millimeters; SD: standard deviation; VA: visual acuity; CVA: corrected visual acuity; UCVA: uncorrected visual acuity; HC: high contrast; LC: low contrast; CS: contrast sensitivity.

**Table 3:** Mean corneal radius differences along the horizontal axis between baseline (before orthokeratology wear) and day 15 for 5 mm and 6 mm OZ lens designs (baseline − 15 d).

Lens design	Parameter (mean ± SD)	Corneal radius (baseline − PRE)								
OZ 5 mm lens	Distance to apex (mm)	−**4.00**	−**3.00**	−**2.00**	−**1.00**	**0.00**	**1.00**	**2.00**	**3.00**	**4.00**
	1 day	−0.04 ± 0.20	0.101 ± 0.19	0.24 ± 0.12	−0.16 ± 0.10	−0.38 ± 0.13	0.07 ± 0.08	0.28 ± 0.09	−0.13 ± 0.27	−0.11 ± 0.40
	7 days	0.02 ± 0.38	0.69 ± 0.29	0.30 ± 0.16	−0.66 ± 0.20	−0.84 ± 0.07	0.11 ± 0.15	0.69 ± 0.19	−0.09 ± 0.39	−0.73 ± 0.82
	15 days	0.15 ± 0.44	0.86 ± 0.36	0.19 ± 0.22	−0.70 ± 0.18	−0.81 ± 0.10	0.00 ± 0.20	0.76 ± 0.27	0.15 ± 0.41	−1.11 ± 1.11

OZ 6 mm lens	Distance to apex (mm)	−**4.00**	−**3.00**	−**2.00**	−**1.00**	**0.00**	**1.00**	**2.00**	**3.00**	**4.00**
	1 day	−0.22 ± 0.36	0.30 ± 0.22	0.13 ± 0.12	−0.18 ± 0.10	−0.28 ± 0.15	−0.04 ± 0.17	0.30 ± 0.12	0.01 ± 0.31	−0.22 ± 0.37
	7 days	−0.17 ± 0.29	0.65 ± 0.43	0.16 ± 0.21	−0.48 ± 0.17	−0.57 ± 0.19	−0.07 ± 0.29	0.53 ± 0.22	0.08 ± 0.59	−0.81 ± 1.14
	15 days	−0.31 ± 1.51	0.77 ± 0.48	0.09 ± 0.26	−0.57 ± 0.31	−0.56 ± 0.12	−0.12 ± 0.28	0.56 ± 0.28	0.18 ± 0.67	−0.95 ± 1.10

Negative values mean flattening and positive values mean steepening. OZ: optical zone; SD: standard deviation.

**Table 4:** Average widths (mm) of optical zone and peripheral rings obtained after 15 days of orthokeratology lenses wear.

	5 mm OZ lens	6 mm OZ lens	*p* value
Nasal PRW	2.3 ± 0.2	1.9 ± 0.1	0.037^*∗*^
OZT	2.8 ± 0.2	3.1 ± 0.1	0.024^*∗*^
Temporal PRW	2.4 ± 0.1	2.2 ± 0.2	0.047^*∗*^

Values are expressed as mean ± SD. SD: standard deviation; OZT: optical zone treatment; PRW: peripheral ring width. ^*∗*^*p* value < 0.05; (Student's *t*-test for paired samples; 5 mm OZ vs. 6 mm OZ).

**Table 5:** Corneal and total spherical aberration measured with Visionix VX110 at 5 mm pupil diameter.

	Corneal Z12 (*µ*m)			Total Z12 (*µ*m)		
Visit	5 mm OZ lens	6 mm OZ lens	*p* value	5 mm OZ lens	6 mm OZ lens	*p* value
PRE	0.144 ± 0.030	0.132 ± 0.029	0.854	0.041 ± 0.054	0.011 ± 0.029	0.456
1 day	0.403 ± 0.083	0.331 ± 0.089	0.526	—	—	—
7 days	0.644 ± 0.101	0.477 ± 0.153	0.027^*∗*^	—	—	—
15 days	0.603 ± 0.116	0.476 ± 0.124	0.039^*∗*^	0.574 ± 0.496	0.451 ± 0.199	0.043^*∗*^

Values are expressed as mean ± SD. SD: standard deviation; OZ: optical zone; Z12: 4^th^ spherical aberration. ^*∗*^*p* < 0.05 comparison between lenses (Student's t-test for paired samples).

## Data Availability

The data used to support the findings of this study are available from the corresponding author upon request.
